# Effects of Albumin Infusion on Serum Levels of Albumin, Proinflammatory Cytokines (TNF-*α*, IL-1, and IL-6), CRP, and MMP-8; Tissue Expression of EGRF, ERK1, ERK2, TGF-*β*, Collagen, and MMP-8; and Wound Healing in Sprague Dawley Rats

**DOI:** 10.1155/2020/3254017

**Published:** 2020-05-20

**Authors:** Arie Utariani, Eddy Rahardjo, David S. Perdanakusuma

**Affiliations:** ^1^Department of Anesthesiology and Reanimation, Dr. Soetomo General Academic Hospital, Faculty of Medicine, Universitas Airlangga, Surabaya, Indonesia; ^2^Department of Plastic Reconstructive & Aesthetic Surgery, Dr. Soetomo General Academic Hospital, Faculty of Medicine, Universitas Airlangga, Surabaya, Indonesia

## Abstract

In this study, we sought to determine the roles of albumin in wound healing, which is infused both pre- and postoperatively in malnourished patients presenting with hypoalbuminemia. For the purposes of the study, we used 25 male Sprague Dawley rats of predetermined weight and age, which were initially maintained in a standard environment and fed the same diet for 7 days prior to being segregated into one of the following five groups: A, control, normal protein feed (20% casein); B, hypoalbuminemia, 25% rat albumin infusion prior to surgery; C, hypoalbuminemia, normal protein feed (20% casein); D, hypoalbuminemia, 25% rat albumin infusion after surgery; and E, hypoalbuminemia, low-protein feed (casein 2%). The animals in all five groups were subjected to four deep incisions in their dorsal muscle fascia. On days 1, 3, 5, and 7 after surgery, ELISA was used to determine serum levels of TNF-*α*, IL-1, IL-6, CRP, and MMP-8, whereas immunohistochemistry was used to determine the tissue expression of EGFR, ERK1, ERK2, TGF-*β*, collagen, and MMP-8. Significant reductions in serum levels of TNF-*α*, IL-1, and CRP were detected in the groups receiving albumin infusion and the high-casein diet (*P* < 0.05). The administration of albumin and a high-casein diet also increased the tissue expression of EGFR, ERK1, ERK2, TGF-*β*, and collagen and decreased that of MMP-8 relative to the hypoalbuminemia control (*P* < 0.05). We propose that the administration of albumin promoted NF-*κ*B signaling which, in turn, induced the transduction and transcription of factors involved in wound healing. Albumin infusion and dietary proteins play vital roles in accelerating the wound healing process, as they can contribute to correcting the hypoalbuminemic state. These findings provide insights that will contribute to our understanding of wound healing, particularly in malnourished patients.

## 1. Introduction

Among the total population, the prevalence of malnutrition lies between 30% and 50%, with the prevalence being higher (85%) in the long-term facilities [1]. Malnourished patients with protein deficiency have a high risk of infection, impaired wound healing, and prolonged hospitalization. Moreover, Yu et al. identified a link between hypoalbuminemia and a high risk of acute kidney injury and mortality [2]. Hypoalbuminemia is a condition associated with a deficiency in albumin caused by a reduction in protein intake, and its prevalence is related to patient age and gender, comorbidities, and dietary intake. Among elderly patients in Brazil, the prevalence of hypoalbuminemia can be as high as 90%, and a similar percentage (89%) has been reported from a hospital in Nepal [3, 4]. In this regard, albumin deficiency is known to prolong the inflammatory phase, reduce fibroblast numbers, hinder proteoglycan and collagen biosynthesis, impede neoangiogenesis, and has detrimental effects on wound shape [5].

Albumin is the dominant plasma protein (50%–60%) and plays an important role in maintaining osmotic pressure at the required 75%–80%. The amounts of albumin synthesized can vary according to the clinical condition [6]. In hypoalbuminemic patients with adequate nutritional intake and optimal liver function, thyroid hormones and cortisol are released to promote the synthesis of albumin mRNA and protein, and albumin production is regulated by feedback loops. Albumin induces the expression of EGFR by activating ERK1/2 and upregulating NF-*κ*B [7, 8], and in rats, it has been demonstrated that increases in EGFR levels are associated with accelerated corneal epithelialization [9, 10]. EGFR play an important role in wound healing process through stimulating tyrosine kinase activity that activates gene transcriptions, DNA synthesis, and cell proliferation [11]. That is why activation of EGFR will have an impact on ERK activity, which is important in regulating cell growth, differentiation, proliferations, migration, and spreading [12]. EGFR also plays an important role in regulating TGF-*β* expression, which plays an important role in wound healing: inflammation, stimulating angiogenesis, fibroblast proliferation, collagen synthesis, and deposition and remodelling of the new extracellular matrix through the SMAD pathway [13]. Another protein that has an impact on the wound healing process is MMP-8, whose overexpression in chronic wound plays a detrimental role in wound repair through activating the collagenase type 1 activity [14]. The hypoalbuminemic state is characterized by increases in the acute-phase proteins CRP, TNF-*α*, IL-1, and IL-6, which are associated with enhanced morbidity and mortality [7, 15–17]. The increased number of proinflamatory cytokines due to the hypoalbuminemic state can upregulate i-Nos production, cause premature fibroblast senescence, and delay skin epithelial proliferation [18]. Therefore, a significant relationship has been detected between preoperative albumin levels and the duration of postoperative wound healing [19–21]. The hypoalbuminemic state is also associated with prolonged inflammation, tissue edema, elevated levels of reactive oxygen species, muscle wasting, and an increase in mortality [22].

Currently, there is comparatively little information available regarding the relationship between serum albumin levels and wound healing in malnourished patients. Moreover, the roles of albumin in the transduction of the genes governing wound healing in hypoalbuminemic patients are unclear. In this study, we sought to establish the role of albumin in regulating the transduction factors and proteins that can affect wound healing in hypoalbuminemia. To this end, we examined the roles of albumin in the wound healing process and in determining the expression of EGFR, ERK1/ERK2, TGF-*β*, and MMP-8 proteins and fasting serum protein levels. We anticipate that the findings of this study will make a valuable contribution toward developing appropriate therapeutic approaches for the oral or parenteral treatment of hypoalbuminemia patients undergoing surgery worldwide.

## 2. Materials and Methods

The research protocol for this study was approved by the Ethics Committee of the Faculty of Veterinary Medicine, Universitas Airlangga, Surabaya, Indonesia. The study was conducted between April and October 2009 at the Biochemistry Laboratorium, Faculty of Medicine, Universitas Brawijaya, and Pathology and Anatomy Laboratory, Faculty of Medicine, Universitas Airlangga, Surabaya, Indonesia.

This study had a posttest, control-only group design, and as study animals, we used healthy male Sprague Dawley rats aged 3 months and weighing 250–300 g (supplier: Cellular and Molecular Biology Laboratory, Gadjah Mada University). The sample size was calculated using the Federer Formula, according to which the number of rats allocated to each treatment group should be at least three. However, to maintain an adequate sample size in the event of death (≥20%), the number of rats in each group was increased to five. The rats were allocated to one of the following five groups: A, control, normal protein feed (20% casein according to AIN 93); B, hypoalbuminemia, 25% rat albumin infusion prior to surgery; C, hypoalbuminemia, normal protein feed (20% casein); D, hypoalbuminemia, 25% rat albumin infusion after surgery; and E, hypoalbuminemia, low-protein feed (casein 2%).

**Figure 1:**
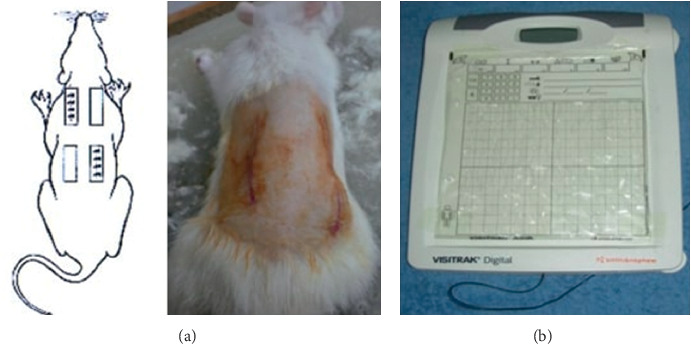
(a) Incision location. (b) Visitrek.

For the first 7 days of the trial, all animals were fed a standard diet of 5 g 100 g^−1^·BW·d^−1^·rat^−1^ and their weight was measured daily. The rats in groups B, C, D, and E were then fed a low-protein diet (casein 2%) for 14 days to induce a hypoalbuminemic state characterized by a 20% decrease in body weight. For all animals, we initially determined the serum levels of albumin, CRP, IL-1, IL-6, and TNF-*α*. For group B, 25% intravenous rat albumin was administered at 1 mL 100 g^−1^·BW for 2 days until normal albumin levels (>2.7 g·dL^−1^) were attained. Group C rats were fed a normal protein diet (5 g 100 g^−1^·BW·d^−1^) for 7–14 days to correct the hypoalbuminemia. Surgery comprised four 2-cm-deep incisions in the dorsal muscle fascia of the musculus spinotrapezius and musculus external oblique (Figure 1(a)). Three hours after the operation, group D rats were administered 25% rat albumin serum (1 mL 100 g^−1^·BW·d^−1^) for 2 days, whereas the rats in group E were maintained in the hypoalbuminemic state. Wound area, serum albumin, CRP, IL-1, IL-6, and TNF-*α* levels, and tissue expression of EGFR, ERK1/ERK2, TGF-*β*, MMP-8, and collagen were measured on days 1, 3, 5, and 7 after surgery. Wound area (length (mm) × width (mm)) was measured using VISITRAK™ (Smith and Nephew, London, UK) (Figure 1(b)). ELISA (Quantikine ELISA Kits) was used to measure the levels of serum albumin, CRP, IL-1, IL-6, and TNF-*α* according to kit instructions, whereas to confirm the expression of EGFR, ERK1/2, TGF-*β*, MMP-8, and collagen, Dako immunostaining Kits were used. Preparation for immunohistochemistry started with dehydrated specimens using alcohol, fixated with formalin 10% before made into a paraffin block. After a day, the paraffin was cut using a rotary microtome with 4–6 *μ*m thickness. The specimens were washed and incubated with Dako immunohistochemistry kit (1 : 100) for 60 minutes. Mayer Hematoxilen was used as the counterstaining agent. There were totally 5 specimens for each groups (control and experiment groups) with a confident interval of 90% and *P* value of 80%. Protein expressions were quantified manually by 2 blinded pathologists, using a light microscope with 400x magnification, in 20 HPF.

Sample distribution and homogeneity were tested using the Kolmogorov–Smirnov *Z* normality test and Tukey HSD homogeneity tests. Significant differences between pairs of group means were determined by ANOVA. Significant differences were interpreted with a *P* value less than 0.05.

## 3. Results

### 3.1. Characteristics of Research Subjects

Tukey HSD homogeneity tests results revealed similarities in the initial body weights and albumin concentrations among the rats in all groups (*P*=0.459; *P*=0.129), and Kolmogorov–Smirnov *Z* normality tests indicated that the initial body weights and albumin concentrations were normally distributed (*P*=0.761; *P*=0.490).

### 3.2. Effects of a Presurgical Low-Protein Diet

The effects of the low-protein diet, evaluated using ELISA, revealed that there were significant reductions in serum albumin in the low-protein groups compared with the control group (*P*=0.003; Table 1). In contrast, there were significant increases in TNF-*α*, IL-1, and CRP in the low-protein groups relative to the control group (*P* < 0.05). Moreover, the IL-6 and MMP-8 levels in the low-protein groups were higher than those in the control group, although the differences were not significant (*P* > 0.05).

### 3.3. Effects of Preoperative Albumin Infusion and Normal Protein Diet on the Hypoalbuminemia Group

Hotelling's *t* [2] analysis (Table 2) revealed significant differences among the presurgical albumin infusion (B), presurgical normal protein diet (C), and hypoalbuminemia (D and E) diet groups in terms of serum levels of albumin, TNF-*α*, IL-1, IL-6, CRP, and MMP-8 (*P*=0.01). There were significant increases in plasma albumin in groups B (preoperative albumin infusion) and C (normal protein diet) compared with groups D and E (hypoalbuminemia) (ANOVA; *P*=0.01), whereas serum TNF-*α*, IL-1, and CRP levels in groups B and C were significantly reduced compared with those in groups D and E (ANOVA; *P*=0.01).

### 3.4. Effects of Pre- and Postsurgical Albumin Infusion on the Hypoalbuminemia Groups

Table 3 shows that there were no significant differences between the preoperative (B) and postoperative (D) albumin infusions groups in terms of the serum levels of albumin, TNF-*α*, IL-1, IL-6, CRP, and MMP-8. Similarly, there were no significant differences among the pre- and postoperative albumin infusion groups and the normal protein diet groups in terms of their serum TNF-*α*, IL-1, IL-6, MMP-8, and CRP levels (*P* > 0.05).  Table 4 indicates a significant increase in serum IL-1 levels in the low-protein diet group (E) (*P* < 0.05) compared with those in the control (A), preoperative albumin infusion (B), postoperative albumin infusion (D), and normal protein diet (C) groups. Moreover, we detected no significant differences in serum CRP levels among the hypoalbuminemia, preoperative albumin infusion (B), postoperative albumin infusion (D), and normal protein diet (C) groups (*P* > 0.05).

### 3.5. Immunohistochemical Analysis

**Figure 2:**
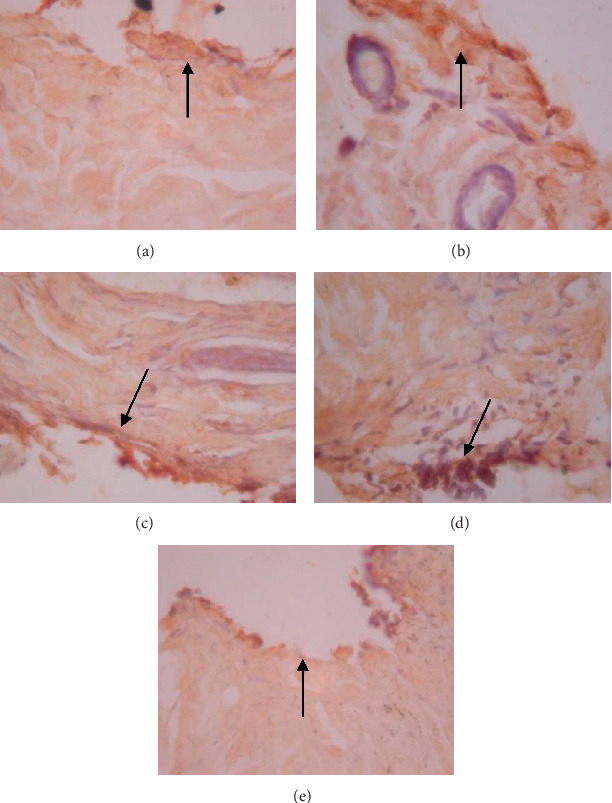
Expression of ERK1 on 5 wound tissue specimens in groups A, B, C, D, and E at the 5^th^ day. *Note*. Brown color represented the expression of ERK1 phospho (pointed out with the black arrow), while transparency showed that there was not any ERK1 expression.

**Figure 3:**
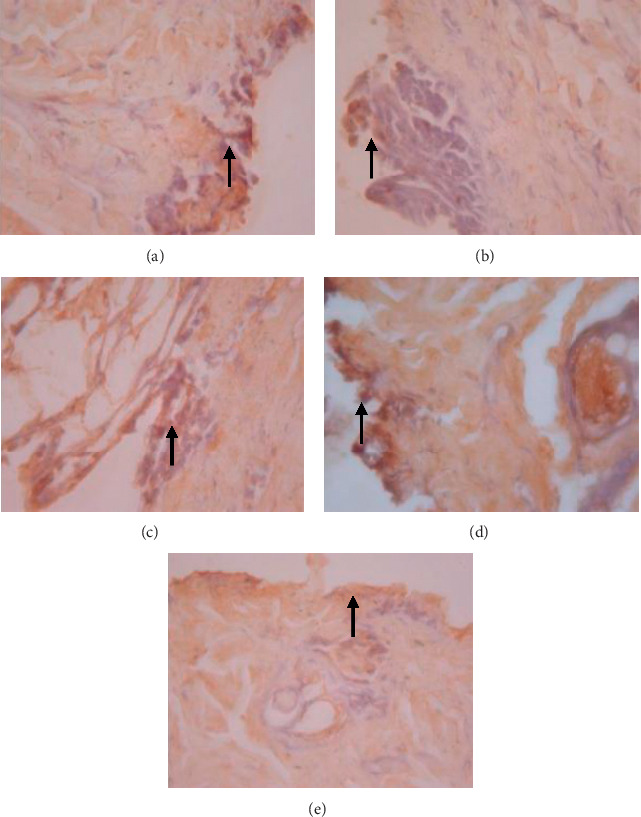
Expression of ERK2 on 5 wound tissue specimens in groups A, B, C, D, and E at the 5^th^ day. *Note*. Brown color represented the expression of ERK2 phospho (pointed out with the black arrow), while transparency showed that there was not any ERK2 expression.

**Figure 4:**
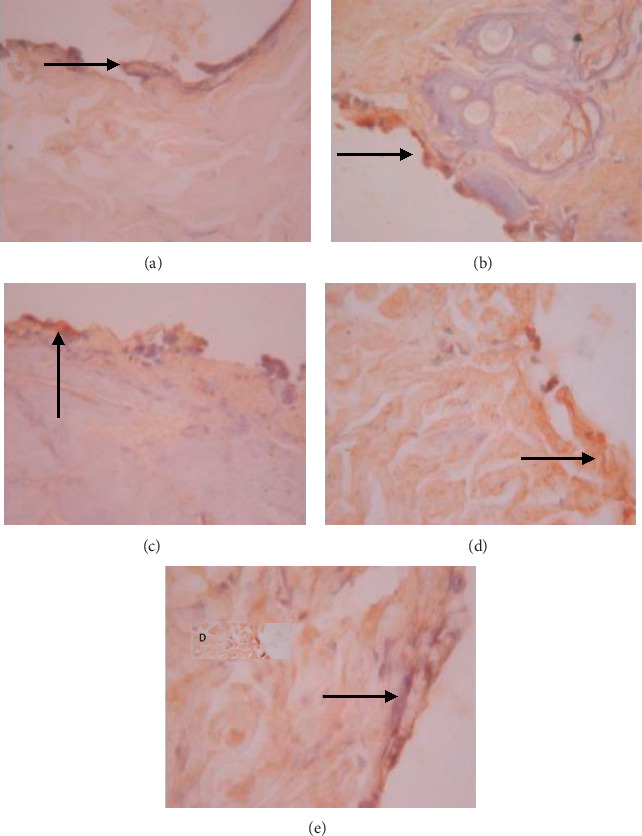
Expression of TGF-*β* on 5 wound tissue specimens in groups A, B, C, D, and E at the 5^th^ day. *Note*. Brown color represented the expression of TGF-*β* phospho (pointed out with the black arrow), while transparency showed that there was not any TGF-*β* expression.

**Figure 5:**
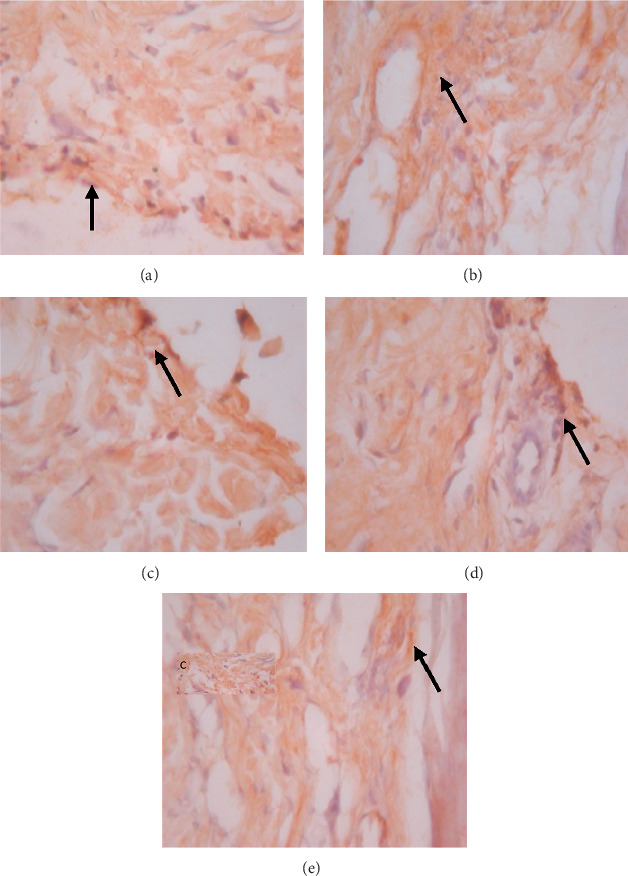
Expression of MMP-8 on 5 wound tissue specimens in groups A, B, C, D, and E at the 5^th^ day. *Note*. Brown color represented the expression of MMP-8 phospho (pointed out with the black arrow), while transparency showed that there was not any MMP-8 expression.

The data presented in Table 5 indicate that preoperative albumin infusion (group B), normal protein diet (group C), and postoperative albumin infusion (group D) all promoted an upregulation of EGFR, ERK1, ERK2, TGF-*β*, and collagen expression relative to the that in the hypoalbuminemia group (group E). Compared with group E rats, the expression of EGFR in group B, C, and D rats had increased significantly by postoperative day 3 (*P* < 0.05), whereas the expression of ERK1, ERK2, TGF-*β*, and collagen had significantly increased by day 5 after surgery (*P* < 0.05) (Figures 2–4). Furthermore, we detected a significant reduction in the expression of MMP-8 in group B, C, and D rats compared with that in group E rats, particularly at postoperative day 5 and thereafter (Figure 5).

### 3.6. Effects of EGFR, IL-1, IL-6, TGF-*β*, Collagen, and MMP-8 Expression on Wound Area at Days 1, 3, 5, and 7 after Surgery

**Figure 6:**
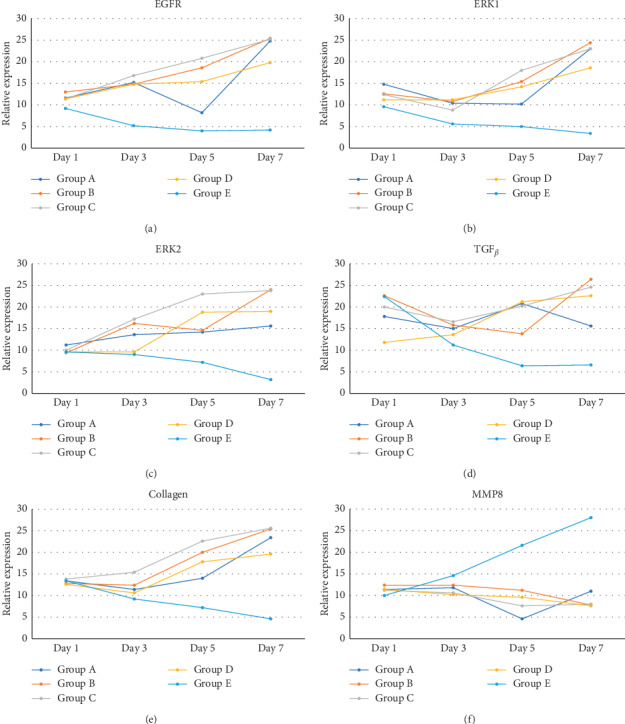
Graphs of EGFR (a), ERK1 (b), ERK2 (c), TGF-*β* (d), collagen (e), and MMP-8 (f) and wound tissue on day 1, 3, 5, and 7 in groups A, B, C, D, and E.

**Figure 7:**
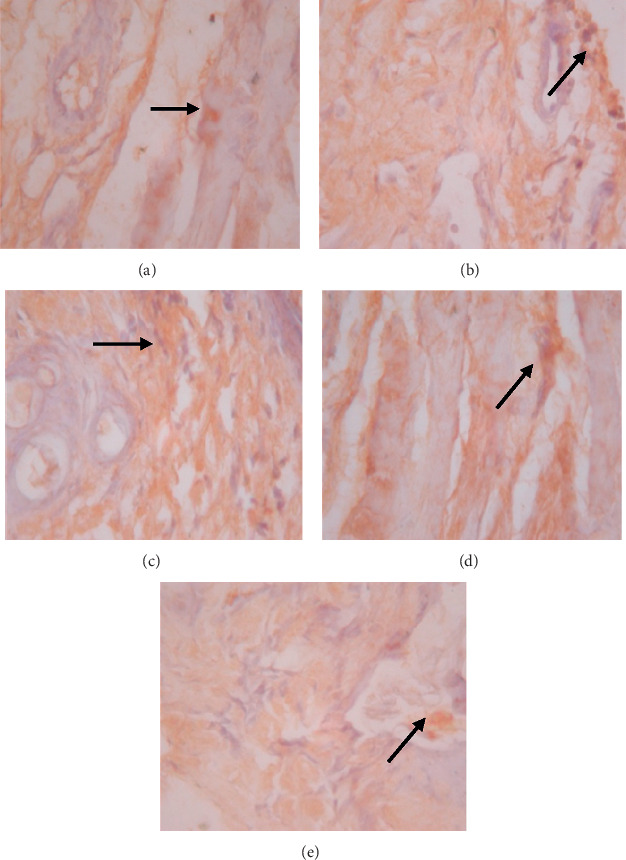
Expression of collagen on 5 wound tissue specimens in groups A, B, C, D, and E at the 5^th^ day. *Note*. Brown color represented the expression of collagen phospho (pointed out with the black arrow), while transparency showed that there was not any collagen expression.

As shown in Figure 6(a), we detected significant increases in EGFR expression in groups B, C, and D from day 3 postsurgery (*P* < 0.05), whereas in contrast, there were significant daily decreases in EGFR expression in group E (*P* < 0.05). Similarly, as shown in Figure 6(b), ERK1 was significantly upregulated by day 5 after surgery in groups B, C, and D, whereas postoperative ERK1 levels decreased steadily from day 5 onwards (Figure 2). Consistently, we observed increases in mean ERK2 expression in groups B, C, and D from postoperative day 5, whereas for group E, ERK2 expression showed a continuous reduction commencing on postoperative day 3 and decreased significantly (*P* < 0.01) from postoperative day 5 onwards (Figures 6(c) and 3).  Figure 6(c) also indicates that there were significant increases in TGF-*β* expression in groups B, C, and D on postoperative day 3, but significant (*P* < 0.01) daily postoperative decreases in group E rats. Likewise, significant (*P* < 0.01) increases in collagen expression were detected in groups B, C, and D by postoperative day 5, whereas collagen expression showed a steady decline each day after surgery in group E (Figures 6(d) and 7). In contrast to the aforementioned trends, we noted significant (*P* < 0.01) decreases in MMP-8 expression in groups B, C, and D commencing on day 5 after surgery, whereas there was a significant continuous increase in MMP-8 expression (*P* < 0.01) in group E following the operation (Figure 5).

VISITRAK™ measurements revealed that by day 3 after surgery, the wound area in the preoperative albumin infusion group (B) was comparable to that in control group A. Accelerated wound healing was also detected in the normal protein diet group (C) by day 5 after surgery, and by day 7, the wound area of rats in this group was equal to that in group A and B rats. Similarly, by day 7 postsurgery, the wound area of the postoperative albumin infusion group (D) was the similar to that in the control and preoperative albumin infusion groups. Comparably, we found that wound healing in the group receiving a normal protein diet (C) was faster than that in groups B, D, and A. In contrast, the wound areas of the hypoalbuminemia group (E) remained wide until day 7 after surgery, and the wound closure rate in these rats was slower than that in the rats of groups A to D.

## 4. Discussion

It has previously been demonstrated that albumin infusion in patients with hypoalbuminemia may accelerate wound healing by modulating the expression of transduction factors and proteins [19–21]. The hypoalbuminemic state is known to affect the expression of acute-phase proteins such as TNF-*α*, IL-1, IL-6, and CRP, all of which play important roles in tissue damage [16, 17]. Moreover, albumin is associated with the transcription of various tissue-forming proteins controlled by EGFR, ERK1, and ERK2 expression, the upregulation of which activates the NF-*κ*B pathway and thereby accelerates wound healing.

In the present study, we observed that there were significant differences between the animals receiving a normal protein diet (20% casein) and those being fed a low-protein diet (2% casein), with hypoalbuminemia being observed in the latter group (Table 1). Pre- and postoperative albumin infusion and normal protein nutrition were administered in the attempt to correct the hypoalbuminemia, and we found that these treatments significantly increased serum albumin levels (Table 3). Moreover, we found that the increase in serum albumin levels in the pre- and postsurgery albumin infusion groups was significantly greater than that for the normal protein diet group (Table 5). These findings are consistent with those reported previously, [23] which indicated that rats on a low-protein diet showed a downregulation of albumin mRNA expression and reduced albumin gene transcription. Similarly, Marten et al. found that albumin gene transcription was reduced in the liver cells of rats with hepatoma, in which amino acid intake and uptake were restricted [17, 24].

The hypoalbuminemia seen in the 2% casein diet group can be attributed to significant increases in the levels of the acute-phase proteins TNF-*α*, IL-1, IL-6, and CRP compared with the controls (Table 1). However, after pre- and postsurgical albumin infusion or administration of a normal protein diet, the levels of TNF-*α*, IL-1, IL-6, CRP, and MMP-8 were significantly reduced relative to the hypoalbuminemia group (Table 3). These findings corroborate with those of an earlier study reporting that malnutrition causes an increase in proinflammatory cytokines [25]. Buck et al. revealed that upregulated expression of TNF-*α* and IL-1 may occur in response to anorexia and muscle and fat catabolism [26], and Crevel et al. stated that increases in the levels of TNF-*α* and other proinflammatory cytokines inhibit albumin biosynthesis in cachexia [27]. Thus, malnutrition can enhance the inflammatory response, aggravates hypoalbuminemia, and increases patient morbidity and mortality.

Our immunohistochemical analysis of EGFR revealed no significant differences between the low-protein diet (2% casein) and control (20% casein) groups on the first day after surgery. The graph shown in Figure 6(a) reveals that EGFR expression was lower in the low-protein diet group than the control group. Similarly, Repertinger et al. reported no significant differences in EGFR expression between the null and wild-type groups they studied [28]. Given the findings of previous studies, we assume that, under normal circumstances, cell proliferation begins on the first day after surgery, which is consistent with the initial phase of wound healing [29–31]. Even under circumstances of protein deficiency, cell proliferation may still commence immediately in response to trauma and naturally decline thereafter.

The observed reduction in EGFR expression in the low-protein diet group was followed by significant decreases in ERK1/ERK2 expression relative to that in the control (Table 5; Figure 6). EGFR downregulation in response to dietary protein deprivation and hypoalbuminemia is known to suppress the activation of mitogen-activated protein kinase (MAPK), which regulates ERK1 and ERK2 expression. The latter two proteins play important roles in cell division and the control of signaling processes and substrate phosphorylation in the cytosol and nucleus. Perturbation of ERK1/ERK2 expression decreases NF-*κ*B pathway activation, which, in turn, disrupts TGF-*β* expression and stimulation of collagen-secreting fibroblasts. In the present study, the hypoalbuminemia group was characterized by significantly less TGF-*β* and collagen than the control group (Table 5; Figure 6). We also detected upregulated expression of MMP-8 and delayed wound repair in the hypoalbuminemia group (Table 5; Figure 6). In this regard, Nwoweh et al. stated that as MMP-8 is a proteolytic enzyme that degrades collagen and other cellular matrices, its upregulation may impede wound healing [32].

In the present study, rats receiving albumin infusion and a normal protein diet showed significant elevations in the expression of EGFR, ERK1, ERK2, TGF-*β*, and collagen. Moreover, these treatments also suppressed tissue MMP-8 expression (Figure 6). Previously, it has be shown that albumin induces 223 genes associated with the regulation of EGFR, [8] an increase in the expression of which upregulates ERK1 and ERK2 and subsequently activates the NF-kB pathway which, in turn, increases the expression of TGF-*β* and collagen. The reduction in the expression of MMP-8 we observed in tissues can be attributed a decrease in inflammation and an increase in collagen production in response to elevated serum albumin levels [25].

**Figure 8:**
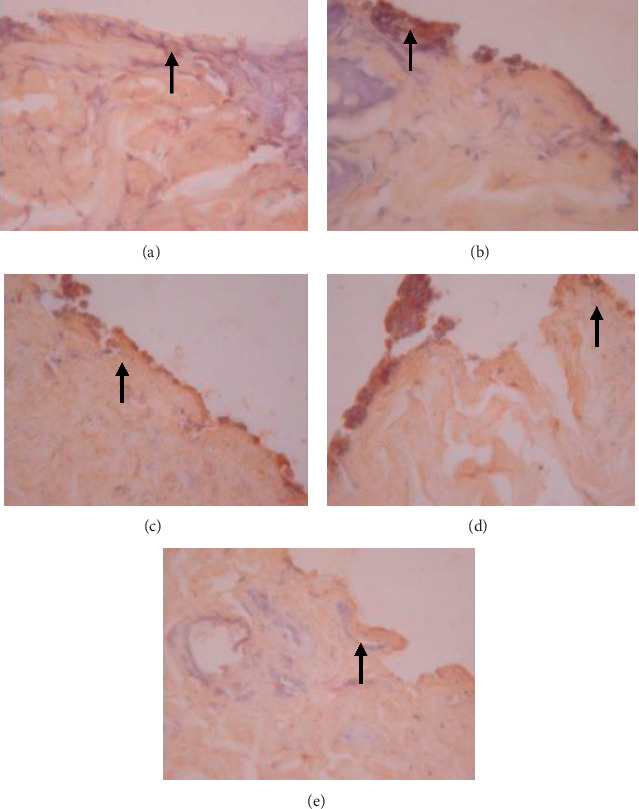
Expression of EGFR phospho on 5 wound tissue specimens in groups A, B, C, D, and E at the 5^th^ day. *Note*. Brown color represented the expression of EGFR phospho (pointed out with the black arrow), while transparency showed that there was not any EGFR expression.

We observed in the present study that the levels of EGFR, ERK1, ERK2, TGF-*β*, collagen, and MMP-8 were altered in the wound area and injured tissues on days 1, 3, 5, and 7 after surgery. There was a significant increase in EGFR expression on day 3 followed by increases in ERK1, ERK2, TGF-*β*, and collagen on day 5 after surgery (Table 5; Figures 6(a)–6(e); Figures 2–4, 8). In contrast, MMP-8 expression had decreased significantly by postoperative day 5 compared with the hypoalbuminemia group (Table 5; Figures 6(f) and 5). These findings are consistent with those reported by Repertinger et al., who observed that EGFR levels had increased significantly in wild-type rats by day 5 after surgery and that at day 3 following the operation, the angiogenic activity of EGFR was significantly higher in wild-type rats than in null rats [28].

**Figure 9:**
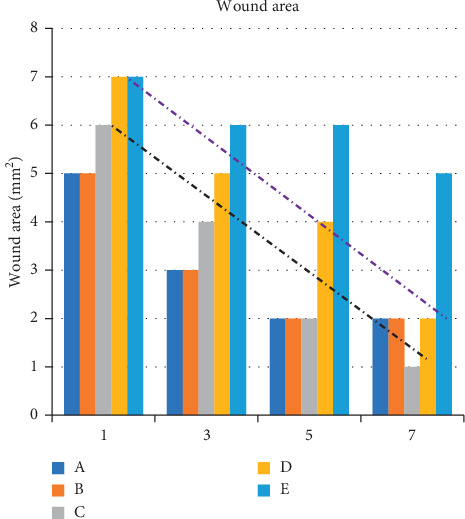
Graph of wound healing processes in group A, B, C, D, and E after surgery.

Our measurement of wound areas using VISITRAK™ (Figure 9) revealed that by day 3 after surgery, the wound area in the preoperative albumin infusion groups was similar to that recorded in control rats and also that by postoperative day 5, the wound area in the normal protein diet group was comparable to that in the preoperative albumin infusion and control groups. Moreover, by day 7 after surgery, the wound area was smallest in the normal protein diet group, whereas the pre- and postoperative albumin infusion groups and the control group did not differ significantly from each other in terms of wound area. The wound healing rates increased concomitantly with increases in the expression levels of EGFR, ERK1, ERK2, TGF-*β*, and collagen, all of which had increased significantly as of postoperative days 3 and 5. These observations are consistent with those reported by Repertinger et al., who found that re-epithelialization commenced on day 3 after surgery and was complete by postoperative day 5 [28].

Albumin infusion and normal protein diet administration in hypoalbuminemic rats before or after injury may increase EGFR activity, which, in turn, induces ERK1/ERK2 signals, activates the NF-*κ*B pathway, and subsequently upregulates the expression of albumin, TGF-*β*, MMP-8, and collagen. Moreover, albumin administration may downregulate expression of the proinflammatory cytokines TNF-*α*, IL-1, and IL-6, as well as CRP and MMP-8. These modes of action explain the observed acceleration of wound healing by day 3 and peak wound healing rate by day 5 after surgery for the albumin infusion and normal protein diet groups relative to the hypoalbuminemia group.

We believe that the findings of this study may provide new insights that will advance our understanding of the role of albumin administration in the wound healing process, as well as providing a scientific basis for the development of albumin supplementation therapy for presurgery hypoalbuminemia patients in daily practice.

## 5. Conclusion

The administration of albumin infusion and normal protein diet to hypoalbuminemic rats before or after surgical insertion was demonstrated to increase the activity of EGFR, which, in turn, induces ERK1/ERK2 signaling that subsequently activates NF-*κ*B pathways associated with the expression of proteins such as albumin, TGF-*β*, MMP-8, and collagen. In addition, administration of albumin was also shown to reduce the expression of proinflammatory cytokines (TNF-*α*, IL1, and IL-6), CRP, and MMP-8. Compared with the group of rat that remained in a hypoalbuminemic state after the injury, both of these responses in the rats receiving albumin infusion and a normal protein diet were found to contribute to the acceleration of the wound healing process observed by the 3^rd^ day after surgery, which peaked on the 5^th^ day.

## Figures and Tables

**Table 1:** Comparison of mean albumin, TNF-*α*, IL-1, IL-6, CRP, and MMP-8 for the control (A) and the hypoalbuminemia groups.

Variable (ng·mL^−1^)	Mean (±SD)		*P*
	Group		
	A	LP diet (hypoalbuminemia)	
Albumin	2.82 ± 0.52	1.77 ± 0.53	0.003^*∗*^
TNF-*α*	5.23 ± 1.35	10.56 ± 0.49	0.007^*∗*^
IL-1	26.36 ± 6.63	52.57 ± 2.65	0.007^*∗*^
1L-6	17.02 ± 6.46	28.71 ± 7.50	0.172
CRP	26.32 ± 6.73	53.77 ± 3.20	0.008^*∗*^
MMP-8	63.61 ± 23.57	108.38 ± 28.83	0.169

Analysis was performed using one-way ANOVA.

**Table 2:** Comparison of mean albumin, TNF-*α*, IL-1, IL-6, CRP, and MMP-8 for the hypoalbuminemia + albumin infusion (B), hypoalbuminemia + normal protein diet (C), and hypoalbuminemia groups (D and E).

Variable	Group (mean ± SD)					HT
	B	C	D and E (hypoalbuminemia)	Total	*P*	*P*
Albumin	5.48 ± 1.30	4.03 ± 0.99	2.30 ± 1.38	3.93 ± 1.38	0.01^*∗*^	0.01^*∗*^
TNF-*α*	2.92 ± 1.98	4.32 ± 1.52	9.29 ± 1.43	5.84 ± 2.96	0.01^*∗*^	
IL-1	19.81 ± 9.76	21.79 ± 7.46	46.09 ± 6.94	29.23 ± 14.48	0.01^*∗*^	
IL-6	20.03 ± 2.60	19.96 ± 5.66	21.22 ± 4.105	20.40 ± 4.00	0.87	
MMP-8	75.34 ± 11.33	74.86 ± 22.36	79.99 ± 16.28	76.73 ± 16.15	0.87	
CRP	19.65 ± 9.97	21.67 ± 7.65	46.26 ± 6.84	29.19 ± 14.68	0.01^*∗*^	

HT: Hotelling's trace.

**Table 3:** Comparison of mean albumin, TNF-*α*, IL-1, IL-6, CRP, and MMP-8 for presurgical albumin infusion (B) and postsurgical albumin infusion (D) groups.

Variable (ng·mL^−1^)	Mean (±SD)		*P*
	Group		
	B	D	
Albumin	5.48 ± 1.31	5.81 ± 2.22	0.888
TNF-*α*	6.27 ± 1.11	6.93 ± 1.64	0.878
IL-1	3.1 ± 5.41	3.45 ± 8.01	0.902
IL-6	15.89 ± 4.11	17.13 ± 4.31	0.980
CRP	31.58 ± 5.57	34.71 ± 8.14	0.895
MMP-8	59.33 ± 16.17	66.00 ± 14.13	0.934

Analysis was performed using one-way ANOVA.

**Table 4:** Comparison of mean TNF-*α*, IL-1, IL-6, MMP-8, and CRP for postoperative (A) control, (B) hypoalbuminemia + preoperative albumin infusion, (C) hypoalbuminemia + normal protein diet, (D) hypoalbuminemia + postoperative albumin infusion, and hypoalbuminemia (E) groups.

Variable	Group (mean ± SD)							HT
	A	B	C	D	E	Total	*P*	*P*
TNF-*α*	2.77 ± 0.78	6.27 ± 1.12	5.84 ± 1.14	6.94 ± 1.64	10.54 ± 0.57	6.46 ± 2.74	0.01^*∗*^	0.01^*∗*^
IL-1	13.88 ± 3.81	31.66 ± 5.36	29.27 ± 5.47	34.52 ± 8.09	52.40 ± 2.63	32.35 ± 13.50	0.01^*∗*^	
IL-6	18.33 ± 8.17	15.23 ± 4.44	31.09 ± 33.47	18.69 ± 10.57	13.30 ± 5.47	19.33 ± 16.34	0.50	
MMP-8	68.76 ± 31.89	66.19 ± 20.72	60.38 ± 27.43	69.90 ± 41.08	49.33 ± 25.35	62.91 ± 28.53	0.81	
CRP	13.62 ± 3.95	31.53 ± 5.64	29.45 ± 5.55	35.27 ± 7.63	52.89 ± 2.80	32.43 ± 13.55	0.01^*∗*^	

HT: Hotelling's trace.

**Table 5:** Comparison of mean EGFR, ERK1, ERK2, TGF-*β*, collagen, and MMP-8 for control (A), hypoalbuminemia + presurgical albumin infusion (B), hypoalbuminemia + normal protein diet (C), hypoalbuminemia + infusion postoperative albumin (D), and hypoalbuminemia (E) on days 1, 3, 5, and 7 after surgery.

Variable	Day	Group (mean ± SD)							HT
		A	B	C	D	E	Total	*P*	*P*
EGFR	1	11.60 ± 3.64	13.00 ± 1.58	11.40 ± 2.19	11.40 ± 2.30	9.20 ± 1.48	11.32 ± 2.49	0.20	0.01^*∗*^
ERK1		14.80 ± 4.32	12.60 ± 2.60	12.40 ± 2.30	11.20 ± 2.38	9.60 ± 1.14	12.12 ± 3.06	0.81	
ERK2		11.20 ± 1.92	9.40 ± 1.82	10.00 ± 2.45	9.60 ± 1.82	9.60 ± 1.52	9.96 ± 1.88	0.59	
TGF-*β*		17.80 ± 6.26	22.60 ± 2.61	20.00 ± 5.70	11.80 ± 1.92	22.40 ± 2.88	18.92 ± 5.61	0.01^*∗*^	
Collagen		13.40 ± 3.13	12.80 ± 2.59	13.80 ± 2.77	12.60 ± 2.80	13.40 ± 3.43	13.20 ± 2.73	0.96	
MMP-8		11.40 ± 3.97	12.40 ± 2.51	11.20 ± 3.35	11.40 ± 1.67	10.00 ± 1.58	11.28 ± 2.65	0.75	
EGFR	3	15.20 ± 5.16	14.80 ± 1.92	16.8 ± 4.55	14.80 ± 3.83	5.20 ± 3.27	13.36 ± 3.54	0.01^*∗*^	0.01^*∗*^
ERK1		10.40 ± 2.30	10.80 ± 2.39	8.80 ± 3.56	11.20 ± 2.49	5.60 ± 2.40	9.36 ± 3.21	0.02^*∗*^	
ERK2		13.60 ± 2.07	16.20 ± 1.30	17.20 ± 1.92	9.60 ± 1.14	9.00 ± 1.87	13.12 ± 3.75	0.01^*∗*^	
TGF-*β*		15.00 ± 2.91	15.80 ± 3.56	16.60 ± 3.65	13.60 ± 3.05	11.20 ± 2.39	14.44 ± 3.46	0.97	
Collagen		11.40 ± 2.30	12.40 ± 3.29	15.40 ± 3.36	10.60 ± 1.14	9.20 ± 1.30	11.80 ± 3.09	0.01^*∗*^	
MMP-8		11.80 ± 2.39	12.40 ± 2.07	10.60 ± 2.51	10.20 ± 1.30	14.60 ± 2.79	11.92 ± 2.61	0.05^*∗*^	
EGFR	5	8.20 ± 1.64	18.60 ± 3.29	20.80 ± 2.59	15.40 ± 4.33	4.00 ± 1.87	13.4 ± 7.00	0.01^*∗*^	0.01^*∗*^
ERK1		10.20 ± 1.92	15.4 ± 3.78	18.00 ± 3.16	14.20 ± 3.49	5.00 ± 2.55	12.56 ± 5.41	0.01∗	
ERK2		14.20 ± 4.32	14.60 ± 3.58	23.00 ± 3.67	18.80 ± 4.66	7.20 ± 2.17	15.56 ± 6.38	0.01^*∗*^	
TGF		20.80 ± 1.48	13.80 ± 3.35	20.20 ± 2.39	21.20 ± 1.30	6.40 ± 2.30	16.48 ± 6.20	0.01^*∗*^	
Collagen		14.00 ± 2.91	20.00 ± 1.58	22.60 ± 2.70	17.80 ± 5.63	7.20 ± 2.68	16.32 ± 6.28	0.01^*∗*^	
MMP-8		4.60 ± 2.70	11.20 ± 2.17	7.60 ± 2.70	9.80 ± 2.17	21.60 ± 1.95	10.96 ± 6.27	0.01^*∗*^	
EGFR	7	24.80 ± 3.27	25.40 ± 2.96	25.20 ± 4.32	19.80 ± 2.86	4.20 ± 1.79	19.88 ± 8.76	0.01^*∗*^	0.01^*∗*^
ERK1		23.00 ± 3.53	24.40 ± 2.70	23.00 ± 3.00	18.60 ± 1.95	3.40 ± 1.52	18.48 ± 8.30	0.01^*∗*^	
ERK2		15.60 ± 4.83	24.00 ± 2.35	23.80 ± 3.70	19.00 ± 5.66	3.20 ± 1.30	17.12 ± 8.57	0.01^*∗*^	
TGF-*β*		15.60 ± 1.95	26.40 ± 3.36	24.60 ± 3.65	22.60 ± 3.51	6.60 ± 2.07	19.16 ± 7.91	0.01^*∗*^	
Collagen		23.40 ± 3.65	25.40 ± 1.67	25.60 ± 2.51	19.60 ± 1.14	4.60 ± 2.07	19.72 ± 8.31	0.01^*∗*^	
MMP-8		11.00 ± 2.35	7.800 ± 1.30	8.00 ± 2.24	28.00 ± 2.74	28.00 ± 2.74	16.56 ± 9.84	0.01^*∗*^	

HT: Hotelling's trace.

## Data Availability

The statistical analysis data that were used to support the findings of this study are included within the supplementary information files.

## Abbreviations

ANOVA: Analysis of variance
CRP: C-reactive protein
EGFR: Epidermal growth factor receptor
ELISA: Enzyme-linked immunosorbent assay
ERK1: Extracellular signal-related kinase 1
ERK2: Extracellular signal-related kinase 2
HSD: Honestly significant difference
IL-1, IL-6: Interleukin-1, Interleukin-6
MANOVA: Multivariate analysis of variance
MAPK: Mitogen-activated protein kinase
MMP-8: Matrix metalloproteinase-8
NF-*κ*B: Nuclear factor kappa-light-chain-enhancer of activated B cells
TGF-*β*: Transforming growth factor beta
TNF-*α*: Tumor necrosis factor alpha.

## Additional Points

The present study has certain limitations, the most prominent of which is the fact that we have not identified a specific albumin receptor or its role in the wound healing process, and thus, further research is warranted in this regard. Furthermore, the width and the depth of the incision wounds examined in this study were limited in extent, and thus, further research examining wider and deeper incision wounds would be desirable for comparative purposes.

## Supplementary Materials

Supplementary Materials 1. Analysis of albumin level and body weight before surgery: describe the body weight data frequencies in each group before the surgery; describe the albumin level data frequencies in each group before the surgery. 2. Plasma measurement result. 3. Immunohistochemistry measurement result.
