# Supplementary material for: Effects of Albumin Infusion on Serum Levels of Albumin, Proinflammatory Cytokines (TNF-α, IL-1, and IL-6), CRP, and MMP-8; Tissue Expression of EGRF, ERK1, ERK2, TGF-β, Collagen, and MMP-8; and Wound Healing in Sprague Dawley Rats
Source: Int J Inflam. 2020 May 20;2020:3254017. doi: 10.1155/2020/3254017 (PMC7256723; doi:10.1155/2020/3254017)
Supplement: Supplementary Materials — 1. Analysis of albumin level and body weight before surgery: describe the body weight data frequencies in each group before the surgery; describe the albumin level data frequencies in each group before the surgery. 2. Plasma measurement result. 3. Immunohistochemistry measurement result. [file 3254017.f1.pdf]

SUPPLEMENTARY FILES

1. **Analysis of albumin level and body weight pre surgery**

Descriptives

Body weight

	N	Mean	Std. Deviation	Std. Error	95% Confidence Interval for Mean		Minimum	Maximum
					Lower Bound	Upper Bound		
Group A	5	245,180	23,7106	10,6037	215,739	274,621	217,0	280,0
Group B	5	304,800	14,7589	6,6004	286,474	323,126	287,5	324,5
Group C	5	342,720	54,3817	24,3202	275,196	410,244	256,1	386,0
Group D	5	325,240	49,3403	22,0656	263,976	386,504	267,0	387,5
Group E	5	254,040	9,5393	4,2661	242,195	265,885	248,5	270,9
Total	25	294,396	50,9409	10,1882	273,369	315,423	217,0	387,5

Describe the body weight data frequencies in each group before the surgery

Descriptives

Albumin Level

	N	Mean	Std. Deviation	Std. Error	95% Confidence Interval for Mean		Minimum	Maximum
					Lower Bound	Upper Bound		
Group A	5	3,020	,0837	,0374	2,916	3,124	2,9	3,1
Group B	5	3,060	,4930	,2205	2,448	3,672	2,7	3,9
Group C	5	2,580	,0837	,0374	2,476	2,684	2,5	2,7
Group D	5	2,780	,2950	,1319	2,414	3,146	2,4	3,2
Group E	5	3,120	,4868	,2177	2,516	3,724	2,7	3,7
Total	25	2,912	,3734	,0747	2,758	3,066	2,4	3,9

Describe the Albumine level data frequencies in each group before the surgery

ANOVA

Body Weight

	Sum of Squares	df	Mean Square	F	Sig.
Between Groups	37228,130	4	9307,032	7,430	,001
Within Groups	25051,380	20	1252,569		
Total	62279,510	24			

ANOVA

Albumin Level

	Sum of Squares	df	Mean Square	F	Sig.
Between Groups	4,250	4	1,063	5,477	,004
Within Groups	3,880	20	,194		
Total	8,130	24			

Normality Test Of Body Weight And Albumin Level Pre-Surgery

One-Sample Kolmogorov-Smirnov Test

		Group	Body Weight	Albumin Level
N		25	25	25
Normal Parameters[a,b]	Mean	3,00	294,396	2,912
	Std. Deviation	1,443	50,9409	,3734
Most Extreme Differences	Absolute	,156	,134	,167
	Positive	,156	,134	,167
	Negative	-,156	-,115	-,095
Kolmogorov-Smirnov Z		,779	,670	,834
Asymp. Sig. (2-tailed)		,579	,761	,490

a. Test distribution is Normal.

b. Calculated from data.

Describe the data distribution analysis for body weight and albumin level, both data were normally distributed

Homogenity test for Albumin level and body weight

Body Weight

	Group	N	Subset for alpha = 0.05	
			1	2
Student-Newman-Keuls[a]	Control	5	245,180	
	Hypoalbumin	5	254,040	
	Albumin Infusion pre surgery	5		304,800
	Albumin Infusion post surgery	5		325,240
	Normal Protein Diet pre surgery	5		342,720
	Sig.		,696	,232
Tukey HSD[a]	Control	5	245,180	
	Hypoalbumin	5	254,040	
	Albumin Infusion pre surgery	5	304,800	304,800
	Albumin Infusion post surgery	5		325,240
	Normal Protein Diet pre surgery	5		342,720
	Sig.		,096	,459

Means for groups in homogeneous subsets are displayed.

a. Uses Harmonic Mean Sample Size = 5,000.

Albumin

	Group	N	Subset for alpha = 0.05	
			1	2
Student-Newman-Keuls[a]	Albumin Infusion Pre Surgery	5	1,6600	
	Albumin Infusion Post surgery	5	1,7000	
	Hypoalbumin	5	1,8400	
	Normal Protein Diet presurgery	5	1,8800	
	Control	5		2,7800
	Sig.		,858	1,000
Tukey HSD[a]	Albumin Infusion Pre Surgery	5	1,6600	
	Albumin Infusion Post surgery	5	1,7000	
	Hypoalbumin	5	1,8400	
	Normal Protein Diet presurgery	5	1,8800	
	Control	5		2,7800
	Sig.		,931	1,000

Means for groups in homogeneous subsets are displayed.

a. Uses Harmonic Mean Sample Size = 5,000.

2. Plasma Measurement Result

RAT'S PLASMA MEASUREMENT RESULT POST SURGERY		
**TNFa**		
	Value	Group
0,544	1,885732525	A1
0,753	2,552824769	A2
0,763	2,584743058	A3
0,772	2,613469518	A4
1,212	4,017874242	A5
1,388	4,579636132	D1
1,524	5,013724864	D2
1,551	5,099904245	C1
1,639	5,38078519	C2
1,694	5,55633578	B2-1
1,716	5,626556017	B2-2
1,841	6,025534631	D3
1,857	6,076603894	D4
1,866	6,105330354	C3
1,982	6,475582509	B2-3
2,141	6,983083307	C4
2,302	7,496967763	B2-4
2,31	7,522502394	D5
2,395	7,793807852	C4
2,934	9,514203639	B2-5
2,959	9,593999362	B1-1
3,215	10,41110756	B1-2
3,35	10,84200447	B1-3
3,352	10,84838813	B1-4
3,404	11,01436323	B1-5
**IL6**		
	Value	Group
0,21	5,5	A1
0,227	7,573170732	A2
0,237	8,792682927	A3
0,241	9,280487805	A4
0,244	9,646341463	A5
0,248	10,13414634	D1
0,25	10,37804878	D2
0,26	11,59756098	C1
0,262	11,84146341	C2
0,262	11,84146341	B2-1
0,279	13,91463415	B2-2
0,284	14,52439024	D3

0,293	15,62195122	D4
0,301	16,59756098	C3
0,309	17,57317073	B2-3
0,316	18,42682927	C4
0,328	19,8902439	B2-4
0,336	20,86585366	D5
0,337	20,98780488	C4
0,349	22,45121951	B2-5
0,386	26,96341463	B1-1
0,389	27,32926829	B1-2
0,406	29,40243902	B1-3
0,431	32,45121951	B1-4
0,9	89,64634146	B1-5
**IL1**		
	Value	Group
0,541	9,902555911	A1
0,728	12,88977636	A2
0,742	13,11341853	A3
0,752	13,27316294	A4
1,187	20,22204473	A5
1,372	23,17731629	D1
1,503	25,26996805	D2
1,53	25,70127796	C1
1,657	27,73003195	C2
1,677	28,04952077	B2-1
1,679	28,08146965	B2-2
1,804	30,07827476	D3
1,828	30,46166134	D4
1,83	30,49361022	C3
1,941	32,26677316	B2-3
2,119	35,11022364	C4
2,257	37,31469649	B2-4
2,258	37,33067093	D5
2,36	38,9600639	C4
2,876	47,2028754	B2-5
2,922	47,93769968	B1-1
3,198	52,34664537	B1-2
3,252	53,20926518	B1-3
3,302	54,00798722	B1-4
3,334	54,51916933	B1-5
**MMP8**		
	Value	Group
0,212	18,85714286	A1

0,23	27,42857143	A2
0,239	31,71428571	A3
0,242	33,14285714	A4
0,246	35,04761905	A5
0,251	37,42857143	D1
0,252	37,9047619	D2
0,262	42,66666667	C1
0,263	43,14285714	C2
0,28	51,23809524	B2-1
0,286	54,0952381	B2-2
0,291	56,47619048	D3
0,292	56,95238095	D4
0,302	61,71428571	C3
0,31	65,52380952	B2-3
0,32	70,28571429	C4
0,327	73,61904762	B2-4
0,335	77,42857143	D5
0,338	78,85714286	C4
0,352	85,52380952	B2-5
0,364	91,23809524	B1-1
0,388	102,6666667	B1-2
0,391	104,0952381	B1-3
0,409	112,6666667	B1-4
0,431	123,1428571	B1-5
**CRP**		
	Value	Group
0,544	9,410914928	A1
0,747	12,66934189	A2
0,759	12,86195827	A3
0,769	13,02247191	A4
1,214	20,16532905	A5
1,39	22,99036918	D1
1,556	25,65489567	D2
1,572	25,9117175	C1
1,644	27,06741573	C2
1,69	27,80577849	B2-1
1,715	28,2070626	B2-2
1,837	30,16532905	D3
1,86	30,53451043	D4
1,865	30,61476726	C3
1,981	32,47672552	B2-3
2,143	35,07704655	C4
2,302	37,62921348	B2-4

2,304	37,66131621	D5
2,402	39,23434992	C4
2,913	47,43659711	B2-5
2,936	47,80577849	B1-1
3,151	51,25682183	B1-2
3,318	53,93739968	B1-3
3,334	54,19422151	B1-4
3,336	54,22632424	B1-5

ELISA results of the pro-inflammatory cytokine level in blood plasma

3. **Immunohistochemistry Measurement Result**

Group		IMMUNOHISTOCHEMISTRY MEASUREMENT RESULT					
		COLAGEN-1	MMP-8 active	tgfb1	egfr-phospho	erk 1	erk 2
A	1	26	9	19	8	7	11
	2	20	8	21	7	11	14
	3	21	11	21	7	12	15
	4	17	2	23	8	10	10
	5	11	6	18	6	9	17
	6	16	9	20	11	11	21
B	1	6	21	9	3	5	6
	2	5	25	8	6	2	8
	3	9	20	3	3	9	9
	4	11	21	6	2	5	4
	5	9	20	9	6	5	8
	6	5	21	6	6	4	9
C	1	18	11	11	21	19	20
	2	19	10	13	17	14	15

	**3**	21	15	15	15	15	14
	**4**	20	10	19	17	10	10
	**5**	15	13	11	21	11	12
	**6**	22	10	11	23	19	14
**D**	**1**	21	6	20	20	16	21
	**2**	20	8	19	24	18	29
	**3**	25	4	22	21	20	20
	**4**	26	9	23	17	22	24
	**5**	20	8	18	19	20	23
	**6**	21	11	17	22	14	21

**MEAN OF IMMUNOHISTOCHEMISTRY MEASUREMENT RESULT**						**Group**
**COLAGEN-1**	**MMP-8 active**	**tgfb1**	**egfr-phospho**	**erk1**	**erk2**	
**14**	**5**	**20**	**8**	**10**	**15**	**A**
**8**	**21**	**7**	**4**	**5**	**7**	**B**
**19**	**12**	**13**	**19**	**15**	**14**	**C**
**22**	**8**	**20**	**21**	**18**	**23**	**D**

IMMUNOHISTOCHEMISTRY ANALYSIS

Group	Day	colagen	MMP8	TGFb1	EGFRp	ERK1	ERK2
Group A	1	12	12	11	10	12	10
		15	10	12	9	14	9
		10	9	20	18	10	14

		18	18	26	10	17	12
		11	11	21	12	19	9
		12	8	20	11	21	11
		10	11	23	9	11	12
		15	10	24	7	10	10
		18	9	20	9	9	9
		10	8	19	11	8	8
		11	11	20	10	14	12
Group E		14	12	26	10	10	9
		10	11	11	9	12	9
		12	10	12	10	10	8
		10	12	10	12	15	11
Group D		15	14	15	15	12	12
		10	12	12	14	10	9
		16	10	11	11	9	8
		10	12	20	15	10	11
		12	15	26	12	13	8
		15	10	24	14	14	10
		16	10	23	13	16	11
		14	9	19	14	17	10
Group B		11	15	20	11	10	7
		15	12	18	10	15	10
		12	14	15	15	9	6
		10	10	22	12	14	12
		17	6	16	10	12	10
		20	9	18	9	10	10
Group C		15	14	29	10	12	12
Group A	3	10	9	20	19	9	14
		12	10	15	14	14	15
		15	12	14	10	8	16
		9	15	13	22	10	12
		12	11	17	15	9	10
		11	13	13	11	11	11
		9	12	10	9	3	6
		8	18	10	2	5	9
		11	14	15	5	9	11
		10	12	9	8	7	10
Group E		7	15	10	4	5	16
Group D		8	17	12	2	4	9
		11	11	12	12	9	9
		10	9	10	10	11	8
		12	12	13	15	12	11

		9	10	15	18	15	10
		8	9	11	11	10	15
		11	9	18	19	9	10
		10	11	19	15	10	18
		18	14	15	17	10	16
		12	12	17	12	9	15
		10	10	10	16	15	15
		9	11	16	13	12	17
Group B		12	15	18	14	10	17
		19	9	20	18	9	18
		10	15	19	15	5	16
		15	10	15	10	10	20
		17	9	18	19	6	15
		12	10	16	14	11	19
Group C		16	10	11	22	14	17
Group A	5	12	5	19	8	7	11
		10	5	21	7	11	14
		15	4	21	7	12	15
		17	2	23	8	10	10
		11	6	18	6	9	17
		16	9	20	11	11	21
		6	21	9	3	5	6
		5	25	8	6	2	8
		9	20	3	3	9	9
		11	21	6	2	5	4
		9	20	9	6	5	8
Group E		5	21	6	6	4	9
		15	9	21	19	12	20
		10	8	20	10	10	15
		19	13	23	16	16	24
Group D		20	8	22	12	14	13
		12	9	19	17	11	25
		25	11	20	20	19	22
		18	11	11	21	19	20
		19	10	13	17	14	15
		21	15	15	15	15	14
		20	10	19	17	10	10
		15	13	11	21	11	12
Group B		22	10	11	23	19	14
Group C		21	6	20	20	16	21
		20	8	19	24	18	29
		25	4	22	21	20	20

		26	9	23	17	22	24
		20	8	18	19	20	23
		21	11	17	22	14	21
**Group A**		22	11	18	25	20	20
		25	9	15	29	29	12
		20	15	17	24	21	15
		29	10	13	26	23	10
		23	9	20	26	25	23
	**7**	21	10	15	20	22	21
		3	25	9	6	3	3
		5	26	7	3	5	5
		2	28	5	2	2	4
		6	32	4	6	5	2
		5	20	9	8	6	9
**Group E**		7	29	8	4	2	2
		20	9	19	16	16	18
		21	8	23	18	18	10
		20	7	28	20	20	25
**Group D**		19	5	24	23	21	20
		23	9	26	24	16	23
		18	5	20	22	18	22
		25	6	25	26	26	25
		24	9	26	28	28	20
		26	7	22	21	24	26
		28	8	31	26	23	24
		30	5	20	25	26	19
**Group B**		24	9	28	24	21	25
		22	11	19	20	20	23
		26	5	25	32	23	20
**Group C**		26	9	26	24	26	29
		25	8	24	25	26	21
		25	6	20	20	29	20
		29	7	29	26	20	26

Descriptives

TNFα (ng/mL)

	N	Mean	Std. Deviation	Std. Error	95% Confidence Interval for Mean		Minimum	Maximum
					Lower Bound	Upper Bound		
A	5	2,73093	,780620	,349104	1,76166	3,70020	1,886	4,018
B	5	6,27258	1,119976	,500869	4,88195	7,66322	5,100	7,794
C	5	5,84360	1,139342	,509529	4,42892	7,25828	4,580	7,523
D	5	6,93393	1,642551	,734571	4,89443	8,97343	5,556	9,514
E	5	10,54197	,575074	,257181	9,82792	11,25602	9,594	11,014
Total	25	6,46460	2,744709	,548942	5,33164	7,59756	1,886	11,014

Describe TNFα Plasma level data frequencies in each group

Descriptives

IL1 (ng/mL)

	N	Mean	Std. Deviation	Std. Error	95% Confidence Interval for Mean		Minimum	Maximum
					Lower Bound	Upper Bound		
A	5	13,88019	3,807165	1,702616	9,15297	18,60741	9,903	20,222
B	5	31,59904	5,419591	2,423715	24,86973	38,32835	25,701	38,960
C	5	29,26358	5,481073	2,451210	22,45793	36,06923	23,177	37,331
D	5	34,58307	8,016305	3,585001	24,62951	44,53662	28,050	47,203
E	5	52,40415	2,628987	1,175719	49,13983	55,66847	47,938	54,519
Total	25	32,34601	13,497256	2,699451	26,77461	37,91740	9,903	54,519

Describe IL1 Plasma level data frequencies in each group

Descriptives

IL6 (ng/mL)

	N	Mean	Std. Deviation	Std. Error	95% Confidence Interval for Mean		Minimum	Maximum
					Lower Bound	Upper Bound		
A	5	8,15854	1,679654	,751164	6,07297	10,24410	5,500	9,646
B	5	15,89024	4,115199	1,840373	10,78055	20,99994	11,598	20,988
C	5	14,30488	4,405799	1,970333	8,83436	19,77540	10,134	20,866
D	5	17,13415	4,313868	1,929220	11,77777	22,49052	11,841	22,451
E	5	29,03659	2,179825	,974847	26,32998	31,74320	26,963	32,451
Total	25	16,90488	7,658621	1,531724	13,74355	20,06620	5,500	32,451

Describe IL6 Plasma level data frequencies in each group

Descriptives

CRP (ng/mL)

	N	Mean	Std. Deviation	Std. Error	95% Confidence Interval for Mean		Minimum	Maximum
					Lower Bound	Upper Bound		
A	5	13,62600	3,949461	1,766253	8,72210	18,52991	9,411	20,165
B	5	31,58106	5,573215	2,492418	24,66100	38,50112	25,912	39,234
C	5	29,40128	5,595341	2,502312	22,45375	36,34882	22,990	37,661
D	5	34,71108	8,146043	3,643021	24,59643	44,82572	27,806	47,437
E	5	52,28411	2,795750	1,250297	48,81273	55,75549	47,806	54,226
Total	25	32,32071	13,574840	2,714968	26,71729	37,92412	9,411	54,226

Describe CRP Plasma level data frequencies in each group

Descriptives

MMP8 (ng/mL)

	N	Mean	Std. Deviation	Std. Error	95% Confidence Interval for Mean		Minimum	Maximum
					Lower Bound	Upper Bound		
A	5	29,23810	6,445304	2,882428	21,23519	37,24100	18,857	35,048
B	5	59,33333	16,176465	7,234335	39,24760	79,41907	42,667	78,857
C	5	53,23810	16,541708	7,397677	32,69885	73,77734	37,429	77,429
D	5	66,00000	14,134116	6,320969	48,45018	83,54982	51,238	85,524
E	5	106,76190	11,917136	5,329505	91,96483	121,55898	91,238	123,143
Total	25	62,91429	28,532089	5,706418	51,13682	74,69175	18,857	123,143

Describe MMP-8 Plasma level data frequencies in each group

ANOVA

TNFa (ng/mL)

	Sum of Squares	df	Mean Square	F	Sig.
Between Groups	156,040	4	39,010	31,508	,000
Within Groups	24,762	20	1,238		
Total	180,802	24			

ANOVA

IL1 (ng/mL)

	Sum of Squares	df	Mean Square	F	Sig.
Between Groups	3791,897	4	947,974	32,670	,000
Within Groups	580,325	20	29,016		
Total	4372,222	24			

ANOVA

IL6 (ng/mL)

	Sum of Squares	df	Mean Square	F	Sig.
Between Groups	1157,594	4	289,399	23,141	,000
Within Groups	250,113	20	12,506		
Total	1407,707	24			

ANOVA

CRP (ng/mL)

	Sum of Squares	df	Mean Square	F	Sig.
Between Groups	3814,067	4	953,517	31,337	,000
Within Groups	608,564	20	30,428		
Total	4422,631	24			

ANOVA

MMP8 (ng/mL)

	Sum of Squares	df	Mean Square	F	Sig.
Between Groups	15863,365	4	3965,841	21,585	,000
Within Groups	3674,558	20	183,728		
Total	19537,923	24			

Multiple Comparisons

TNFa (ng/mL)
Tukey HSD

					95% Confidence Interval	
(I) GROUP	(J) GROUP	Mean Difference (I-J)	Std. Error	Sig.	Lower Bound	Upper Bound
A	B	-3,541653*	,703733	,001	-5,64749	-1,43582
	C	-3,112672*	,703733	,002	-5,21850	-1,00684
	D	-4,203000*	,703733	,000	-6,30883	-2,09717
	E	-7,811044*	,703733	,000	-9,91688	-5,70521
B	A	3,541653*	,703733	,001	1,43582	5,64749
	C	,428982	,703733	,972	-1,67685	2,53481
	D	-,661347	,703733	,878	-2,76718	1,44449
	E	-4,269390*	,703733	,000	-6,37522	-2,16356
C	A	3,112672*	,703733	,002	1,00684	5,21850
	B	-,428982	,703733	,972	-2,53481	1,67685
	D	-1,090329	,703733	,544	-3,19616	1,01550
	E	-4,698372*	,703733	,000	-6,80420	-2,59254
D	A	4,203000*	,703733	,000	2,09717	6,30883
	B	,661347	,703733	,878	-1,44449	2,76718
	C	1,090329	,703733	,544	-1,01550	3,19616
	E	-3,608043*	,703733	,000	-5,71388	-1,50221
E	A	7,811044*	,703733	,000	5,70521	9,91688
	B	4,269390*	,703733	,000	2,16356	6,37522
	C	4,698372*	,703733	,000	2,59254	6,80420
	D	3,608043*	,703733	,000	1,50221	5,71388

*. The mean difference is significant at the 0.05 level.

Multiple Comparisons

IL1 (ng/mL)
Tukey HSD

(I) GROUP	(J) GROUP	Mean Difference (I-J)	Std. Error	Sig.	95% Confidence Interval	
					Lower Bound	Upper Bound
A	B	-17,718850*	3,406833	,000	-27,91337	-7,52433
	C	-15,383387*	3,406833	,002	-25,57791	-5,18887
	D	-20,702875*	3,406833	,000	-30,89739	-10,50836
	E	-38,523962*	3,406833	,000	-48,71848	-28,32944
B	A	17,718850*	3,406833	,000	7,52433	27,91337
	C	2,335463	3,406833	,957	-7,85906	12,52998
	D	-2,984026	3,406833	,902	-13,17854	7,21049
	E	-20,805112*	3,406833	,000	-30,99963	-10,61059
C	A	15,383387*	3,406833	,002	5,18887	25,57791
	B	-2,335463	3,406833	,957	-12,52998	7,85906
	D	-5,319489	3,406833	,537	-15,51401	4,87503
	E	-23,140575*	3,406833	,000	-33,33509	-12,94606
D	A	20,702875*	3,406833	,000	10,50836	30,89739
	B	2,984026	3,406833	,902	-7,21049	13,17854
	C	5,319489	3,406833	,537	-4,87503	15,51401
	E	-17,821086*	3,406833	,000	-28,01561	-7,62657
E	A	38,523962*	3,406833	,000	28,32944	48,71848
	B	20,805112*	3,406833	,000	10,61059	30,99963
	C	23,140575*	3,406833	,000	12,94606	33,33509
	D	17,821086*	3,406833	,000	7,62657	28,01561

*. The mean difference is significant at the 0.05 level.

Multiple Comparisons

IL6 (ng/mL)
Tukey HSD

(I) GROUP	(J) GROUP	Mean Difference (I-J)	Std. Error	Sig.	95% Confidence Interval	
					Lower Bound	Upper Bound
A	B	-7,731707*	2,236573	,019	-14,42437	-1,03904
	C	-6,146341	2,236573	,082	-12,83901	,54632
	D	-8,975610*	2,236573	,005	-15,66828	-2,28294
	E	-20,878049*	2,236573	,000	-27,57071	-14,18538
B	A	7,731707*	2,236573	,019	1,03904	14,42437
	C	1,585366	2,236573	,952	-5,10730	8,27803
	D	-1,243902	2,236573	,980	-7,93657	5,44876
	E	-13,146341*	2,236573	,000	-19,83901	-6,45368
C	A	6,146341	2,236573	,082	-,54632	12,83901
	B	-1,585366	2,236573	,952	-8,27803	5,10730
	D	-2,829268	2,236573	,715	-9,52193	3,86340
	E	-14,731707*	2,236573	,000	-21,42437	-8,03904
D	A	8,975610*	2,236573	,005	2,28294	15,66828
	B	1,243902	2,236573	,980	-5,44876	7,93657
	C	2,829268	2,236573	,715	-3,86340	9,52193
	E	-11,902439*	2,236573	,000	-18,59510	-5,20977
E	A	20,878049*	2,236573	,000	14,18538	27,57071
	B	13,146341*	2,236573	,000	6,45368	19,83901
	C	14,731707*	2,236573	,000	8,03904	21,42437
	D	11,902439*	2,236573	,000	5,20977	18,59510

*. The mean difference is significant at the 0.05 level.

Multiple Comparisons

CRP (ng/mL)
Tukey HSD

(I) GROUP	(J) GROUP	Mean Difference (I-J)	Std. Error	Sig.	95% Confidence Interval	
					Lower Bound	Upper Bound
A	B	-17,955056*	3,488737	,000	-28,39466	-7,51545
	C	-15,775281*	3,488737	,002	-26,21489	-5,33567
	D	-21,085072*	3,488737	,000	-31,52468	-10,64547
	E	-38,658106*	3,488737	,000	-49,09771	-28,21850
B	A	17,955056*	3,488737	,000	7,51545	28,39466
	C	2,179775	3,488737	,969	-8,25983	12,61938
	D	-3,130016	3,488737	,895	-13,56962	7,30959
	E	-20,703050*	3,488737	,000	-31,14266	-10,26344
C	A	15,775281*	3,488737	,002	5,33567	26,21489
	B	-2,179775	3,488737	,969	-12,61938	8,25983
	D	-5,309791	3,488737	,561	-15,74940	5,12982
	E	-22,882825*	3,488737	,000	-33,32243	-12,44322
D	A	21,085072*	3,488737	,000	10,64547	31,52468
	B	3,130016	3,488737	,895	-7,30959	13,56962
	C	5,309791	3,488737	,561	-5,12982	15,74940
	E	-17,573034*	3,488737	,001	-28,01264	-7,13343
E	A	38,658106*	3,488737	,000	28,21850	49,09771
	B	20,703050*	3,488737	,000	10,26344	31,14266
	C	22,882825*	3,488737	,000	12,44322	33,32243
	D	17,573034*	3,488737	,001	7,13343	28,01264

*. The mean difference is significant at the 0.05 level.

Multiple Comparisons

MMP8 (ng/mL)
Tukey HSD

(I) GROUP	(J) GROUP	Mean Difference (I-J)	Std. Error	Sig.	95% Confidence Interval	
					Lower Bound	Upper Bound
A	B	-30,095238*	8,572698	,017	-55,74796	-4,44251
	C	-24,000000	8,572698	,074	-49,65272	1,65272
	D	-36,761905*	8,572698	,003	-62,41463	-11,10918
	E	-77,523810*	8,572698	,000	-103,17653	-51,87108
B	A	30,095238*	8,572698	,017	4,44251	55,74796
	C	6,095238	8,572698	,952	-19,55749	31,74796
	D	-6,666667	8,572698	,934	-32,31939	18,98606
	E	-47,428571*	8,572698	,000	-73,08130	-21,77585
C	A	24,000000	8,572698	,074	-1,65272	49,65272
	B	-6,095238	8,572698	,952	-31,74796	19,55749
	D	-12,761905	8,572698	,581	-38,41463	12,89082
	E	-53,523810*	8,572698	,000	-79,17653	-27,87108
D	A	36,761905*	8,572698	,003	11,10918	62,41463
	B	6,666667	8,572698	,934	-18,98606	32,31939
	C	12,761905	8,572698	,581	-12,89082	38,41463
	E	-40,761905*	8,572698	,001	-66,41463	-15,10918
E	A	77,523810*	8,572698	,000	51,87108	103,17653
	B	47,428571*	8,572698	,000	21,77585	73,08130
	C	53,523810*	8,572698	,000	27,87108	79,17653
	D	40,761905*	8,572698	,001	15,10918	66,41463

Descriptives

		N	Mean	Std. Deviation	Std. Error	95% Confidence Interval for Mean		Minimum	Maximum
						Lower Bound	Upper Bound		
ERK1	Group A	6	10.00	1.789	.730	8.12	11.88	7	12
	Group E	6	5.00	2.280	.931	2.61	7.39	2	9
	Group D+B	6	14.67	3.830	1.563	10.65	18.69	10	19
	Group C	6	18.33	2.944	1.202	15.24	21.42	14	22
	Total	24	12.00	5.748	1.173	9.57	14.43	2	22
ERK2	Group A	6	14.67	4.033	1.647	10.43	18.90	10	21
	Group E	6	7.33	1.966	.803	5.27	9.40	4	9
	Group D+B	6	14.17	3.371	1.376	10.63	17.70	10	20
	Group C	6	23.00	3.286	1.342	19.55	26.45	20	29
	Total	24	14.79	6.433	1.313	12.08	17.51	4	29

	N	Mean	Std. Deviation	Std. Error	95% Confidence Interval for Mean		Minimum	Maximum
					Lower Bound	Upper Bound		
Group A	6	7.83	1.722	.703	6.03	9.64	6	11
Group E	6	4.33	1.862	.760	2.38	6.29	2	6
Group D+B	6	19.00	3.098	1.265	15.75	22.25	15	23
Group C	6	20.50	2.429	.992	17.95	23.05	17	24
Total	24	12.92	7.442	1.519	9.77	16.06	2	24

EGFR

Descriptives

TGFb1

	N	Mean	Std. Deviation	Std. Error	95% Confidence Interval for Mean		Minimum	Maximum
					Lower Bound	Upper Bound		
Group A	6	20.33	1.751	.715	18.50	22.17	18	23
Group E	6	6.83	2.317	.946	4.40	9.26	3	9
Group D+B	6	13.33	3.204	1.308	9.97	16.70	11	19
Group C	6	19.83	2.317	.946	17.40	22.26	17	23
Total	24	15.08	6.071	1.239	12.52	17.65	3	23

Descriptives

COLLAGEN

	N	Mean	Std. Deviation	Std. Error	95% Confidence Interval for Mean		Minimum	Maximum
					Lower Bound	Upper Bound		
Group A	6	13.50	2.881	1.176	10.48	16.52	10	17
Group E	6	7.50	2.510	1.025	4.87	10.13	5	11
Group D+B	6	19.17	2.483	1.014	16.56	21.77	15	22
Group C	6	22.17	2.639	1.078	19.40	24.94	20	26
Total	24	15.58	6.234	1.273	12.95	18.22	5	26

Descriptives

MMP8 (ng/mL)

	N	Mean	Std. Deviation	Std. Error	95% Confidence Interval for Mean		Minimum	Maximum
					Lower Bound	Upper Bound		
Group A	5	29.23810	6.445304	2.882428	21.23519	37.24100	18.857	35.048
Group E	5	59.33333	16.176465	7.234335	39.24760	79.41907	42.667	78.857
Group D+B	5	53.23810	16.541708	7.397677	32.69885	73.77734	37.429	77.429
Group C	5	66.00000	14.134116	6.320969	48.45018	83.54982	51.238	85.524
Group A	5	106.76190	11.917136	5.329505	91.96483	121.55898	91.238	123.143
Total	25	62.91429	28.532089	5.706418	51.13682	74.69175	18.857	123.143

ANOVA

EGFR

	Sum of Squares	df	Mean Square	F	Sig.
Between Groups	1164.167	3	388.056	70.770	.000
Within Groups	109.667	20	5.483		
Total	1273.833	23			

ANOVA

COLAGEN

	Sum of Squares	Df	Mean Square	F	Sig.
Between Groups	755.167	3	251.722	36.306	.000
Within Groups	138.667	20	6.933		
Total	893.833	23			

ANOVA

		Sum of Squares	df	Mean Square	F	Sig.
ERK1	Between Groups	601.333	3	200.444	25.266	.000
	Within Groups	158.667	20	7.933		
	Total	760.000	23			
ERK2	Between Groups	740.458	3	246.819	23.340	.000
	Within Groups	211.500	20	10.575		
	Total	951.958	23			

ANOVA

TGFb1

	Sum of Squares	Df	Mean Square	F	Sig.
Between Groups	727.500	3	242.500	40.305	.000
Within Groups	120.333	20	6.017		
Total	847.833	23			

ANOVA

MMP8 (ng/mL)

	Sum of Squares	Df	Mean Square	F	Sig.
Between Groups	15863.365	4	3965.841	21.585	.000
Within Groups	3674.558	20	183.728		
Total	19537.923	24			

Multiple Comparisons

EGFR

Tukey HSD

(I) GROUP	(J) GROUP	Mean Difference (I-J)	Std. Error	Sig.	95% Confidence Interval Lower Bound	95% Confidence Interval Upper Bound
Group A	Group E	3.500	1.352	.076	-.28	7.28
	Group B+D	-11.167*	1.352	.000	-14.95	-7.38
	Group C	-12.667*	1.352	.000	-16.45	-8.88
Group E	Group A	-3.500	1.352	.076	-7.28	.28
	Group B+D	-14.667*	1.352	.000	-18.45	-10.88
	Group C	-16.167*	1.352	.000	-19.95	-12.38
Group B+D	Group A	11.167*	1.352	.000	7.38	14.95
	Group E	14.667*	1.352	.000	10.88	18.45
	Group C	-1.500	1.352	.688	-5.28	2.28
Group C	Group A	12.667*	1.352	.000	8.88	16.45
	Group E	16.167*	1.352	.000	12.38	19.95
	Group B+D	1.500	1.352	.688	-2.28	5.28

*. The mean difference is significant at the 0.05 level.

Multiple Comparisons

Tukey HSD

Dependent Variable	(I) GROUP	(J) GROUP	Mean Difference (I-J)	Std. Error	Sig.	95% Confidence Interval	
						Lower Bound	Upper Bound
ERK1	Group A	Group E	5.000*	1.626	.028	.45	9.55
		Group B+D	-4.667*	1.626	.043	-9.22	-.12
		Group C	-8.333*	1.626	.000	-12.88	-3.78
	Group E	Group A	-5.000*	1.626	.028	-9.55	-.45
		Group B+D	-9.667*	1.626	.000	-14.22	-5.12
		Group C	-13.333*	1.626	.000	-17.88	-8.78
	Group B+D	Group A	4.667*	1.626	.043	.12	9.22
		Group E	9.667*	1.626	.000	5.12	14.22
		Group C	-3.667	1.626	.143	-8.22	.88
	Group C	Group A	8.333*	1.626	.000	3.78	12.88
		Group E	13.333*	1.626	.000	8.78	17.88
		Group B+D	3.667	1.626	.143	-.88	8.22
ERK2	Group A	Group E	7.333*	1.877	.004	2.08	12.59
		Group B+D	.500	1.877	.993	-4.75	5.75
		Group C	-8.333*	1.877	.001	-13.59	-3.08
	Group E	Group A	-7.333*	1.877	.004	-12.59	-2.08
		Group B+D	-6.833*	1.877	.008	-12.09	-1.58
		Group C	-15.667*	1.877	.000	-20.92	-10.41
	Group B+D	Group A	-.500	1.877	.993	-5.75	4.75
		Group E	6.833*	1.877	.008	1.58	12.09
		Group C	-8.833*	1.877	.001	-14.09	-3.58
	Group C	Group A	8.333*	1.877	.001	3.08	13.59
		Group E	15.667*	1.877	.000	10.41	20.92
		Group B+D	8.833*	1.877	.001	3.58	14.09

*. The mean difference is significant at the 0.05 level.

Multiple Comparisons

TGFb1
Tukey HSD

(I) GROUP	(J) GROUP	Mean Difference (I-J)	Std. Error	Sig.	95% Confidence Interval Lower Bound	95% Confidence Interval Upper Bound
Group A	Group E	13.500*	1.416	.000	9.54	17.46
	Group B+D	7.000*	1.416	.000	3.04	10.96
	Group C	.500	1.416	.984	-3.46	4.46
Group E	Group A	-13.500*	1.416	.000	-17.46	-9.54
	Group B+D	-6.500*	1.416	.001	-10.46	-2.54
	Group C	-13.000*	1.416	.000	-16.96	-9.04
Group B+D	Group A	-7.000*	1.416	.000	-10.96	-3.04
	Group E	6.500*	1.416	.001	2.54	10.46
	Group C	-6.500*	1.416	.001	-10.46	-2.54
Group C	Group A	-.500	1.416	.984	-4.46	3.46
	Group E	13.000*	1.416	.000	9.04	16.96
	Group B+D	6.500*	1.416	.001	2.54	10.46

*. The mean difference is significant at the 0.05 level.

Multiple Comparisons

COLLAGEN
Tukey HSD

(I) GROUP	(J) GROUP	Mean Difference (I-J)	Std. Error	Sig.	95% Confidence Interval Lower Bound	95% Confidence Interval Upper Bound
Group A	Group E	6.000*	1.520	.004	1.74	10.26
	Group B+D	-5.667*	1.520	.007	-9.92	-1.41
	Group C	-8.667*	1.520	.000	-12.92	-4.41
Group E	Group A	-6.000*	1.520	.004	-10.26	-1.74
	Group B+D	-11.667*	1.520	.000	-15.92	-7.41
	Group C	-14.667*	1.520	.000	-18.92	-10.41
Group B+D	Group A	5.667*	1.520	.007	1.41	9.92
	Group E	11.667*	1.520	.000	7.41	15.92
	Group C	-3.000	1.520	.231	-7.26	1.26
Group C	Group A	8.667*	1.520	.000	4.41	12.92
	Group E	14.667*	1.520	.000	10.41	18.92
	Group B+D	3.000	1.520	.231	-1.26	7.26

*. The mean difference is significant at the 0.05 level.

Multiple Comparisons

MMP8 (ng/mL)

Tukey HSD

(I) GROUP	(J) GROUP	Mean Difference (I-J)	Std. Error	Sig.	95% Confidence Interval Lower Bound	95% Confidence Interval Upper Bound
Group A	Group B	-30.095238*	8.572698	.017	-55.74796	-4.44251
	Group C	-24.000000	8.572698	.074	-49.65272	1.65272
	Group D	-36.761905*	8.572698	.003	-62.41463	-11.10918
	Group E	-77.523810*	8.572698	.000	-103.17653	-51.87108
Group B	Group A	30.095238*	8.572698	.017	4.44251	55.74796
	Group C	6.095238	8.572698	.952	-19.55749	31.74796
	Group D	-6.666667	8.572698	.934	-32.31939	18.98606
	Group E	-47.428571*	8.572698	.000	-73.08130	-21.77585
Group C	Group A	24.000000	8.572698	.074	-1.65272	49.65272
	Group B	-6.095238	8.572698	.952	-31.74796	19.55749
	Group D	-12.761905	8.572698	.581	-38.41463	12.89082
	Group E	-53.523810*	8.572698	.000	-79.17653	-27.87108
Group D	Group A	36.761905*	8.572698	.003	11.10918	62.41463
	Group B	6.666667	8.572698	.934	-18.98606	32.31939
	Group C	12.761905	8.572698	.581	-12.89082	38.41463
	Group E	-40.761905*	8.572698	.001	-66.41463	-15.10918
Group E	Group A	77.523810*	8.572698	.000	51.87108	103.17653
	Group B	47.428571*	8.572698	.000	21.77585	73.08130
	Group C	53.523810*	8.572698	.000	27.87108	79.17653
	Group D	40.761905*	8.572698	.001	15.10918	66.41463

*. The mean difference is significant at the 0.05 level.

EGFR

Tukey HSD

GROUP	N	Subset for alpha = 0.05	
		1	2
Group E	6	4.33	
Group A	6	7.83	
Group B+D	6		19.00
Group C	6		20.50
Sig.		.076	.688

Means for groups in homogeneous subsets are displayed.

ERK1

Tukey HSD

GROUP	N	Subset for alpha = 0.05		
		1	2	3
Group E	6	5.00		
Group A	6		10.00	
Group B+D	6			14.67
Group C	6			18.33
Sig.		1.000	1.000	.143

Means for groups in homogeneous subsets are displayed.

ERK2

Tukey HSD

GROUP	N	Subset for alpha = 0.05		
		1	2	3
Group E	6	7.33		
Group A	6		14.17	
Group B+D	6		14.67	
Group C	6			23.00
Sig.		1.000	.993	1.000

Means for groups in homogeneous subsets are displayed.

TGFb1

Tukey HSD

GROUP		Subset for alpha = 0.05		
		1	2	3
Group E	6	6.83		
Group B+D	6		13.33	
Group C	6			19.83
Group A	6			20.33

ERK1

Tukey HSD

GROUP	N	Subset for alpha = 0.05		
		1	2	3
Group E	6	5.00		
Group A	6		10.00	
Group B+D	6			14.67
Group C	6			18.33
Sig.		1.000	1.000	.143

Means for groups in homogeneous subsets are displayed.

Sig.		1.000	1.000	.984

Means for groups in homogeneous subsets are displayed.

COLLAGEN

Tukey HSD

GROUP	N	Subset for alpha = 0.05		
		1	2	3
Group E	6	7.50		
Group A	6		13.50	
Group B+D	6			19.17
Group C	6			22.17
Sig.		1.000	1.000	.231

Means for groups in homogeneous subsets are displayed.

MMP8 (ng/mL)

Tukey HSD

Group	N	Subset for alpha = 0.05		
		1	2	3
Group A	5	29.23810		
Group C	5	53.23810	53.23810	
Group B	5		59.33333	
Group D	5		66.00000	
Group E	5			106.76190
Sig.		.074	.581	1.000

Means for groups in homogeneous subsets are displayed.

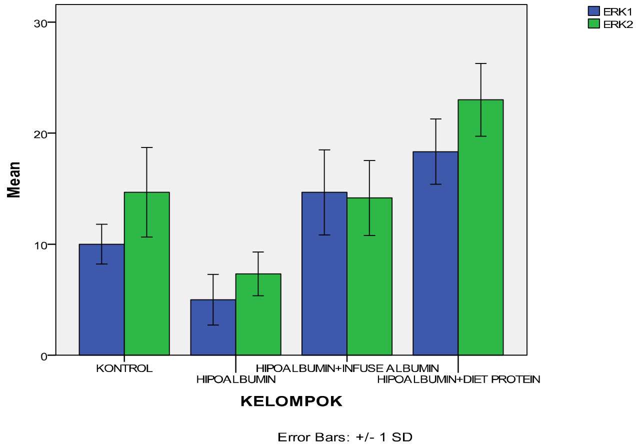

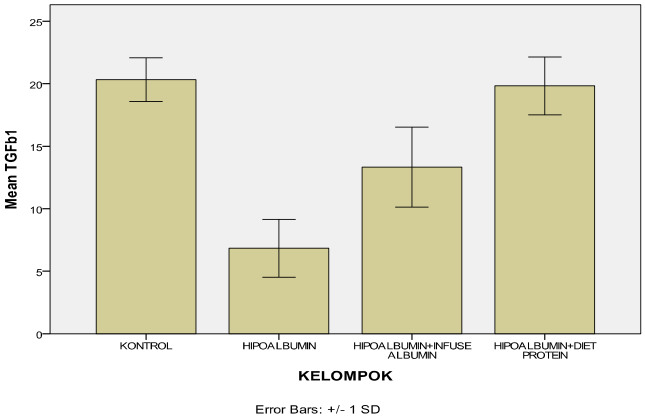

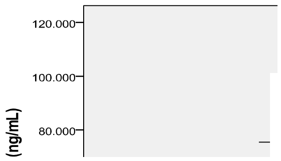

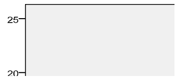

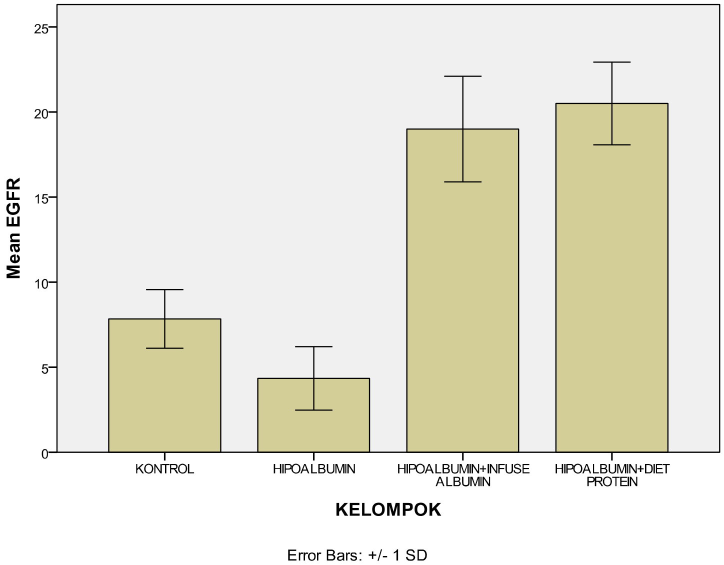

Descriptive Statistics

	kelompok	Mean	Std. Deviation	N
EGFRhr1	1,00	11,6000	3,64692	5
	2,00	13,0000	1,58114	5
	3,00	11,4000	2,19089	5
	4,00	11,4000	2,30217	5
	5,00	9,2000	1,48324	5
	Total	11,3200	2,49533	25
ERK1hr1	1,00	14,8000	4,32435	5
	2,00	12,6000	2,60768	5
	3,00	12,4000	2,30217	5
	4,00	11,2000	2,38747	5
	5,00	9,6000	1,14018	5
	Total	12,1200	3,05941	25
ERK2hr1	1,00	11,2000	1,92354	5
	2,00	9,4000	1,81659	5
	3,00	10,0000	2,44949	5
	4,00	9,6000	1,81659	5
	5,00	9,6000	1,51658	5
	Total	9,9600	1,88149	25
TGFhr1	1,00	17,8000	6,26099	5
	2,00	22,6000	2,60768	5
	3,00	20,0000	5,70088	5
	4,00	11,8000	1,92354	5
	5,00	22,4000	2,88097	5
	Total	18,9200	5,61189	25
Kolagenhr1	1,00	13,4000	3,13050	5
	2,00	12,8000	2,58844	5
	3,00	13,8000	2,77489	5
	4,00	12,6000	2,79285	5
	5,00	13,4000	3,43511	5
	Total	13,2000	2,73861	25
MMP8hr1	1,00	11,4000	3,97492	5
	2,00	12,4000	2,50998	5
	3,00	11,2000	3,34664	5
	4,00	11,4000	1,67332	5
	5,00	10,0000	1,58114	5
	Total	11,2800	2,65393	25

Descriptive Statistics

	kelompok	Mean	Std. Deviation	N
EGFRhr3	1,00	15,2000	5,16720	5
	2,00	14,8000	1,92354	5
	3,00	16,8000	4,54973	5
	4,00	14,8000	3,83406	5
	5,00	5,2000	3,27109	5
	Total	13,3600	5,53685	25
ERK1hr3	1,00	10,4000	2,30217	5
	2,00	10,8000	2,38747	5
	3,00	8,8000	3,56371	5
	4,00	11,2000	2,48998	5
	5,00	5,6000	2,40832	5
	Total	9,3600	3,21299	25
ERK2hr3	1,00	13,6000	2,07364	5
	2,00	16,2000	1,30384	5
	3,00	17,2000	1,92354	5
	4,00	9,6000	1,14018	5
	5,00	9,0000	1,87083	5
	Total	13,1200	3,74522	25
TGFhr3	1,00	15,0000	2,91548	5
	2,00	15,8000	3,56371	5
	3,00	16,6000	3,64692	5
	4,00	13,6000	3,04959	5
	5,00	11,2000	2,38747	5
	Total	14,4400	3,46506	25
Kolagenhr3	1,00	11,4000	2,30217	5
	2,00	12,4000	3,28634	5
	3,00	15,4000	3,36155	5
	4,00	10,6000	1,14018	5
	5,00	9,2000	1,30384	5
	Total	11,8000	3,09570	25
MMP8hr3	1,00	11,8000	2,38747	5
	2,00	12,4000	2,07364	5
	3,00	10,6000	2,50998	5
	4,00	10,2000	1,30384	5
	5,00	14,6000	2,79285	5
	Total	11,9200	2,61279	25

Descriptive Statistics

	kelompok	Mean	Std. Deviation	N
EGFRhr7	1,00	24,8000	3,27109	5
	2,00	25,4000	2,96648	5
	3,00	25,2000	4,32435	5
	4,00	19,8000	2,86356	5
	5,00	4,2000	1,78885	5
	Total	19,8800	8,76223	25
ERK1hr7	1,00	23,0000	3,53553	5
	2,00	24,4000	2,70185	5
	3,00	23,0000	3,00000	5
	4,00	18,6000	1,94936	5
	5,00	3,4000	1,51658	5
	Total	18,4800	8,30723	25
ERK2hr7	1,00	15,6000	4,82701	5
	2,00	24,0000	2,34521	5
	3,00	23,8000	3,70135	5
	4,00	19,0000	5,65685	5
	5,00	3,2000	1,30384	5
	Total	17,1200	8,56991	25
TGFhr7	1,00	15,6000	1,94936	5
	2,00	26,4000	3,36155	5
	3,00	24,6000	3,64692	5
	4,00	22,6000	3,50714	5
	5,00	6,6000	2,07364	5
	Total	19,1600	7,90928	25
Kolagenhr7	1,00	23,4000	3,64692	5
	2,00	25,4000	1,67332	5
	3,00	25,6000	2,50998	5
	4,00	19,6000	1,14018	5
	5,00	4,6000	2,07364	5
	Total	19,7200	8,30923	25
MMP8hr7	1,00	11,0000	2,34521	5
	2,00	7,8000	1,30384	5
	3,00	8,0000	2,23607	5
	4,00	28,0000	2,73861	5
	5,00	28,0000	2,73861	5
	Total	16,5600	9,83650	25

Multivariate Tests[c]

Effect		Value	F	Hypothesis df	Error df	Sig.	Partial Eta Squared
Intercept	Pillai's Trace	,994	422,837[a]	6,000	15,000	,000	,994
	Wilks' Lambda	,006	422,837[a]	6,000	15,000	,000	,994
	Hotelling's Trace	169,135	422,837[a]	6,000	15,000	,000	,994
	Roy's Largest Root	169,135	422,837[a]	6,000	15,000	,000	,994
Group	Pillai's Trace	1,502	1,804	24,000	72,000	,029	,375
	Wilks' Lambda	,086	2,276	24,000	53,539	,006	,458
	Hotelling's Trace	4,743	2,668	24,000	54,000	,001	,542
	Roy's Largest Root	3,347	10,041[b]	6,000	18,000	,000	,770

a. Exact statistic

b. The statistic is an upper bound on F that yields a lower bound on the significance level.

c. Design: Intercept+ Group

Multivariate Tests[c]

Effect		Value	F	Hypothesis df	Error df	Sig.	Partial Eta Squared
Intercept	Pillai's Trace	,995	494,480[a]	6,000	15,000	,000	,995
	Wilks' Lambda	,005	494,480[a]	6,000	15,000	,000	,995
	Hotelling's Trace	197,792	494,480[a]	6,000	15,000	,000	,995
	Roy's Largest Root	197,792	494,480[a]	6,000	15,000	,000	,995
Group	Pillai's Trace	2,471	4,846	24,000	72,000	,000	,618
	Wilks' Lambda	,002	11,533	24,000	53,539	,000	,795
	Hotelling's Trace	44,260	24,896	24,000	54,000	,000	,917
	Roy's Largest Root	37,684	113,053[b]	6,000	18,000	,000	,974

a. Exact statistic

b. The statistic is an upper bound on F that yields a lower bound on the significance level.

c. Design: Intercept+ Group

Multivariate Tests[c]

Effect		Value	F	Hypothesis df	Error df	Sig.	Partial Eta Squared
Intercept	Pillai's Trace	,997	837,612[a]	6,000	15,000	,000	,997
	Wilks' Lambda	,003	837,612[a]	6,000	15,000	,000	,997
	Hotelling's Trace	335,045	837,612[a]	6,000	15,000	,000	,997
	Roy's Largest Root	335,045	837,612[a]	6,000	15,000	,000	,997
Group	Pillai's Trace	2,539	5,215	24,000	72,000	,000	,635
	Wilks' Lambda	,001	14,638	24,000	53,539	,000	,829
	Hotelling's Trace	57,363	32,267	24,000	54,000	,000	,935
	Roy's Largest Root	48,067	144,200[b]	6,000	18,000	,000	,980

a. Exact statistic

b. The statistic is an upper bound on F that yields a lower bound on the significance level.

c. Design: Intercept+ Group

Descriptive Statistics

	Group	Mean	Std. Deviation	N
LLhr1	1,00	4,8000	,83666	5
	2,00	5,4000	1,81659	5
	3,00	5,4000	,89443	5
	4,00	6,6000	2,07364	5
	5,00	6,0000	1,58114	5
	Total	5,6400	1,52425	25
LLhr3	1,00	3,4000	1,14018	5
	2,00	3,4000	1,14018	5
	3,00	4,4000	1,14018	5
	4,00	5,4000	,54772	5
	5,00	6,2000	1,92354	5
	Total	4,5600	1,60935	25
LLhr5	1,00	2,0000	,70711	5
	2,00	2,4000	,89443	5
	3,00	1,8000	,83666	5
	4,00	4,4000	1,14018	5
	5,00	5,8000	1,48324	5
	Total	3,2800	1,86011	25
LLhr7	1,00	2,0000	,70711	5
	2,00	2,6000	1,51658	5
	3,00	1,8000	,83666	5
	4,00	2,4000	1,14018	5
	5,00	5,6000	,89443	5
	Total	2,8800	1,71561	25
